# circSSU72 Promotes Cell Proliferation, Migration and Invasion of Papillary Thyroid Carcinoma Cells by Targeting miR-451a/S1PR2 Axis

**DOI:** 10.3389/fcell.2022.817028

**Published:** 2022-03-14

**Authors:** Zeyu Zhang, Fada Xia, Lei Yao, Bo Jiang, Xinying Li

**Affiliations:** Department of Thyroid Surgery, Xiangya Hospital, Central South University, Changsha, China

**Keywords:** thyroid cancer, papillary thyroid carcinoma, circRNAs, cell proliferation, migration, invasion

## Abstract

**Introduction:** Thyroid cancer is the most common endocrine malignancy with Papillary Thyroid Carcinoma (PTC) as the most common pathological type. Due to low mortality but a high incidence, PTC still causes a relatively heavy burden on financial costs, human health, and quality of life. Emerging researches have indicated that circular RNAs (circRNAs) play a significant regulatory role in various cancers, including PTC. However, the functions and mechanisms of circRNAs derived from SSU72 remain unknown.

**Method:** The expression level of circRNAs derived from the exons of SSU72, miR-361–3p, miR-451a, and S1PR2 was evaluated by qRT-PCR assay or western blot assay. The interactions between circSSU72 (hsa_circ_0009294), miR-451a, and S1PR2 were verified by dual-luciferase reporter assay. Effects of circSSU72, miR-451a, and S1PR2 on cell proliferation, migration, and invasion were confirmed by colony formation assay, cell counting kit-8 (CCK-8), wound healing assay, and Transwell assays *in vitro*.

**Results:** circSSU72 was upregulated in PTC; circSSU72 knockdown inhibited PTC cell proliferation, migration, and invasion. In addition, circSSU72 could negatively regulate miR-451a by functioning as a sponge. circSSU72 promoted PTC cell proliferation, migration, and invasion by targeting miR-451a *in vitro*. We further found that miR-451a inhibited PTC cell proliferation, migration, and invasion by regulating S1PR2. Overall, the circSSU72/miR-451a/S1PR2 axis might influence PTC cell proliferation, migration, and invasion.

**Conclusions:** Overall, circSSU72 (hsa_circ_0009294)/miR-451a/S1PR2 axis may promote cell proliferation, migration, and invasion in PTC. Thus, circSSU72 may serve as a potential biomarker and therapeutic target for PTC.

## Introduction

Thyroid cancer (TC) is the most common endocrine malignancy with papillary thyroid carcinoma (PTC) as the most common pathological type of TC. Although accounting for over 80% TC, PTC usually carries a very good patient prognosis with surgical treatments. (Kim et al., 2020) However, 10–15% of advanced PTC patients suffer from recurrence, and 5–25% from distant metastasis, with radioactive iodine ablation, thyroid-stimulating hormone suppression, and available targeted therapies. (DeGroot et al., 1990; Sebastian et al., 2000; Laha et al., 2020) Due to the low mortality but the high incidence, PTC still causes a relatively heavy burden on financial costs, human health, and quality of life. Thus, studies on oncogenesis and the development of PTC are still needed for more promising therapeutic targets.

Circular RNAs (circRNAs), as a large class of non-coding RNAs, contain a unique covalent loop structure without 5′-cap and 3′-poly (A) structures, resulting in their resistance to exonuclease degradation. circRNAs usually function as competitive endogenous RNAs (ceRNAs) by sponging micro RNAs (miRNAs), therefore influencing mRNAs expression and further oncogenesis and development of diseases. (Xia et al., 2021) Due to their conservation, circRNAs are expected to be valuable biomarkers and therapeutic targets in various cancer types.

SSU72 (SSU72 Homolog, RNA Polymerase II CTD Phosphatase) is a novel phosphatase with dual specificity that can dephosphorylate both phosphoserine/threonine and phosphotyrosine, which is also essential for RNA polymerase II. (Rodríguez-Torres et al., 2013) SSU72 intervenes at different stages of the transcription process by interacting with RNAPII subunits including Rpb2, TFIIB, and other mediators. SSU72 has a unique active site with specific structural characteristics at the C-terminus. It consists of a central 5-stranded β-sheet (β1–β5) enclosed by helices on both sides. SSU72 not only physiologically functions as a cohesin-binding phosphatase, but is also involved in various diseases, including nonalcoholic steatohepatitis, hepatocellular carcinoma, and autoimmune diseases. (Hwang et al., 2021) Moreover, SSU72 shows the highest expression in the thyroid among all the normal tissues. (Fagerberg et al., 2014) Thus, we consider that the SSU72-derived circRNAs may participate in the oncogenesis and development of PTC.

In this study, for the first time, we comprehensively uncovered the biological roles of SSU72-derived circRNAs in oncogenesis and development of PTC, which might provide a potential biomarker and a therapeutic target of PTC.

## Methods

### Patient Samples

30 pairs of PTC tissues and adjacent normal tissues were obtained from PTC patients in the Xiangya Hospital, Central South University from January to June 2020. This study was approved by the Ethics Committee of the Xiangya Hospital and written consent was obtained from all subjects.

### Cell Culture

Human thyroid normal epithelial cell line (Nthy-ori 3-1), and human PTC cell lines (B-CPAP, KTC-1, K1, IHH-4, TPC-1) were purchased from the Shanghai Academy of Sciences. Nthy-ori 3-1 was cultured in Dulbecco’s modified Eagle’s medium (DMEM), while B-CPAP, KTC-1, K1, IHH-4, and TPC-1 in RPMI-1640 medium (Gibco, USA), supplemented with penicillin (100 U/mL), streptomycin (100 μg/ml), and 10% fetal bovine serum (FBS, S Hyclone). All the cell lines were maintained at 37°C with 5% CO2.

### RNA Extraction and Quantitative Real-Time PCR Assay

Total RNAs of thyroid cancer tissues and cells were extracted by using RNAEX reagent (Accurate Biotechnology, Hunan) with instructions of the manufacturer. RNase R (Epicenter Technologies) was used for 15 min at 37°C when RNase R treatment was necessary. The first strand of cDNA was synthesized with Evo M-MLV Mix Kit (Accurate Biotechnology, Hunan). Particularly, the first strand of cDNA of miR-361–3p and miR-451a was synthesized using the stem-loop method, while the first strand of cDNA of U6 was synthesized with a gene-specific primer. SYBR Green Premix Pro Taq HS qPCR Kit (Accurate Biotechnology, Hunan) was used for subsequent qRT-PCR assay on QuantStudio 5 system (ThermoFisher Scientific, USA). The relative expression levels were analyzed using the 2^−ΔΔCt^ method and normalized by beta-actin or U6. The sequences of primers involved in this study were shown in Table 1.

**TABLE 1:** The sequence of primers involved in this study.

Gene		Sequence (5′-3′)
SSU72	Forward	CGA​CAA​GCC​CAA​TGT​TTA​TGA​T
	Reverse	ATC​AAA​CAG​GTC​TTT​GCA​GTT​C
circ_0009293	Forward	CAG​ATG​CTG​CTG​TCA​ATC​CA
	Reverse	TGG​GCT​CAG​TAG​AAG​CAG​AC
circ_0009294	Forward	GTG​TGC​ACT​TCC​CGA​CAT​AC
	Reverse	GGA​ATT​CAG​ATT​GAC​AGC​AGC​A
circ_0009295	Forward	GTG​TGC​ACT​TCC​CGA​CAT​AC
	Reverse	TGT​GTA​TAG​TGA​CAG​CAG​CAT​C
circ_0009296	Forward	GTG​TGC​ACT​TCC​CGA​CAT​AC
	Reverse	GAA​TCC​CCG​TTT​GTG​ACA​GC
circ_0009297	Forward	GTG​TGC​ACT​TCC​CGA​CAT​AC
	Reverse	AGACCCGCACTCCACAAG
miR-361–3p	Forward	GCT​CCC​CCA​GGT​GTG​ATT​C
	Reverse	GTGCAGGGTCCGAGGT
	RT	GTC​GTA​TCC​AGT​GCA​GGG​TCC​GAG​GTA​TTC​GCA​CTG​GAT​ACG​ACA​AAT​CA
miR-451a	Forward	GCG​CAA​ACC​GTT​ACC​ATT​AC
	Reverse	GTGCAGGGTCCGAGGT
	RT	GTC​GTA​TCC​AGT​GCA​GGG​TCC​GAG​GTA​TTC​GCA​CTG​GAT​ACG​ACA​ACT​CA
S1PR2	Forward	CAT​CCT​CCT​TCT​GGA​CTA​TGC
	Reverse	GTG​TAG​ATG​ACG​GGG​TTG​AG
MIF	Forward	GAA​CAA​CTC​CAC​CTT​CGC​CTA​AGA​G
	Reverse	TCT​AAA​CCG​TTT​ATT​TCT​CCC​CAC​CAG
PSMB8	Forward	CTT​TAG​ATG​ACA​CGA​CCC​TAC​C
	Reverse	CAA​TCT​GAA​CGT​TCC​TTT​CTC​C
CAB39	Forward	TGA​GGC​CTT​TCA​CGT​TTT​TAA​G
	Reverse	GGT​TCT​TGA​GGA​GGA​TGT​CTA​G
beta-actin	Forward	CCTGGCACCCAGCACAAT
	Reverse	GGGCCGGACTCGTCATAC
U6	Forward	CTCGCTTCGGCAGCACA
	Reverse	AAC​GCT​TCA​CGA​ATT​TGC​GT

RT, reverse transcription.

### Fluorescence *In Situ* Hybridization Assay

*In situ* hybridization was carried out using probes specific to the circSSU72 sequence. The Nthy-ori 3-1 cell was cultured in a 24-well plate. RNA localization was determined using a FISH kit from RiboBio according to the manufacturer’s protocol. The nucleus was stained using the 4′,6-diamidino-2-phenylindole (DAPI) and the signals were measured by fluorescence microscopy.

### Oligonucleotide Transfection

The hsa_circ_0009294 siRNAs, miR-451a inhibitors, and mimics, as well as negative control (NC), were obtained from Sangon (Shanghai, China). Transfection was performed by Lipofectamine™ RNAi MAX (Invitrogen) according to the instructions of the manufacturer.

### Stable Transfection

Human lentivirus-S1PR2 and lentivirus-hsa_circ_0009294 were purchased from Genechem (Shanghai, China) and transfected into cells using HitransG P (Genechem). Puromycin (Gibco, USA) was used for the selection of cells and green fluorescent protein (GFP) was used to exam the transfection efficiency. The 3’ UTR was contained in the expression vectors for further investigations.

### Dual-Luciferase Activity Assay

The interactions between circSSU72, miR-451a, and S1PR2 were measured by dual-luciferase activity assay in Nthy-ori 3-1 cell. The original sequence of circSSU72 and S1PR2 was constructed into Luc-circSSU72-WT and Luc-S1PR2-WT plasmid, while we mutated the predicted binding site of miR-451a on circSSU72 and S1PR2 in Luc-circSSU72-MUT and Luc-S1PR2-MUT plasmid. Nthy-ori 3-1 cell was cultured in 24-well plates. Plasmids were transfected using X-tremegene HP (ROCHE), while miR-451a mimics and NC mimics were transfected using Lipofectamine™ RNAi MAX (Invitrogen). After incubation of 48 h, relative luciferase activity was detected by a Dual-Luciferase^®^ Reporter Assay System (Promega, Madison, WI) with the renilla luciferase activity as an internal reference.

### Target Prediction of circSSU72 and miR-451a

Three bioinformatics databases were used for target prediction of circSSU72 (hsa_circ_0009294), including Circbank (http://www.circbank.cn/), (Liu et al., 2019) starBase (http://starbase.sysu.edu.cn/), (Li et al., 2014) and CircInteractome (https://circinteractome.irp.nia.nih.gov/). (Dudekula et al., 2016) Meanwhile, TargetScan (http://www.targetscan.org/) (Agarwal et al., 2015) and miRDB (http://mirdb.org/) (Chen and Wang, 2020) were used for target prediction of miR-451a.

### Western Blot Assay

Proteins were extracted using RIPA buffer (Beyotime, Shanghai, China), and the concentration was determined by a BCA kit (ThermoFisher Scientific). An equivalent amount of proteins was isolated by SDS-PAGE, and transferred to polyvinyl fluoride membrane (Merck KGaA). After incubation with primary antibodies overnight at 4°C, and incubation with horseradish peroxidase-conjugated secondary antibodies (FDM007 and FDR007, Fudebio, Hangzhou, China) for 2 h. The membranes were treated with the enhanced chemiluminescent reagents (MILLIPORE, WBKLS0500). The signals were examined by ChemiDox (bio-rad, USA) with the treatment of an enhanced chemiluminescence kit (FD8030, Fudebio, Hangzhou, China). The primary antibodies involved in the present study were GAPDH (1:1000, Abcam), anti-S1PR2 (1:500, Proteintech), anti-AKT (1:1000, Wanleibio), anti-p-AKT (Ser473) (1:1000, Wanleibio).

### Cell Counting Kit-8 Assay

Cell Counting Kit-8 (Beyotime, Shanghai, China) was used to detect cell proliferation ability. An equivalent amount of cells was plated on 96-well plates and CCK8 solution (10 ul/well) was added at pointed time. The absorbance at 450 nm was measured subsequently after 2 h incubation at 37°C.

### 5-Ethynyl-20-Deoxyuridine Incorporation Assay

The EdU assay was performed using a BeyoClick™ EdU Cell Proliferation Kit with Alexa Fluor 555 (Beyotime, Shanghai, China) according to the instructions of the manufacturer.

### Colony Formation Assay

An equivalent amount of TPC-1 and IHH-4 cells were planted into the 6-well plates and incubated for 14 days at 37 °C. After being fixed and stained with 0.1% crystal violet, the colony was counted for comparisons.

### Wound Healing Assay

Cells were cultured in 6-well plates at 37 °C. Scratch wounds were created by using the fine end of 100-μL pipette tips. Images of migrated cells were captured under phase-contrast microscopy at different times.

### Transwell Assay

Transwell assays were used to detect cell migration and invasion and conducted as previously described. (Xia et al., 2020)

### Tumor Formation Assay *In Vivo*

The 6-week old male BALB/c nude mice were purchased from the Department of Laboratory Animal Science, Central South University for the *in vivo* tumor formation assay. TPC-1 cells (1 × 10^6^) that were stably transfected with circSSU72 overexpression vectors or NC vectors were subcutaneously injected into the left armpit of nude mice. Tumor growth was detected at 0, 1, 2, 3, and 4 weeks after injection, and the volume of tumors was recorded as the length×width (DeGroot et al., 1990)×0.5. Four weeks after injection, the mice were euthanized with CO2, and the tumors were collected.

### Statistical Analyses

R 3.3.0 and Statistical Package for Social Sciences 23.0 for Windows (SPSS Inc., Chicago, IL, United States) were used to perform statistical analyses, while GraphPad Prism v7.0 software (GraphPad Software, La Jolla, CA, USA) was used for generating illustrations. One-way analysis of variance (ANOVA) was used for homogeneous variance, while Welch’s ANOVA was applied when the variance was heterogeneous.

## Results

### circSSU72 (hsa_circ_0009294) was Upregulated in PTC

**FIGURE 1:**
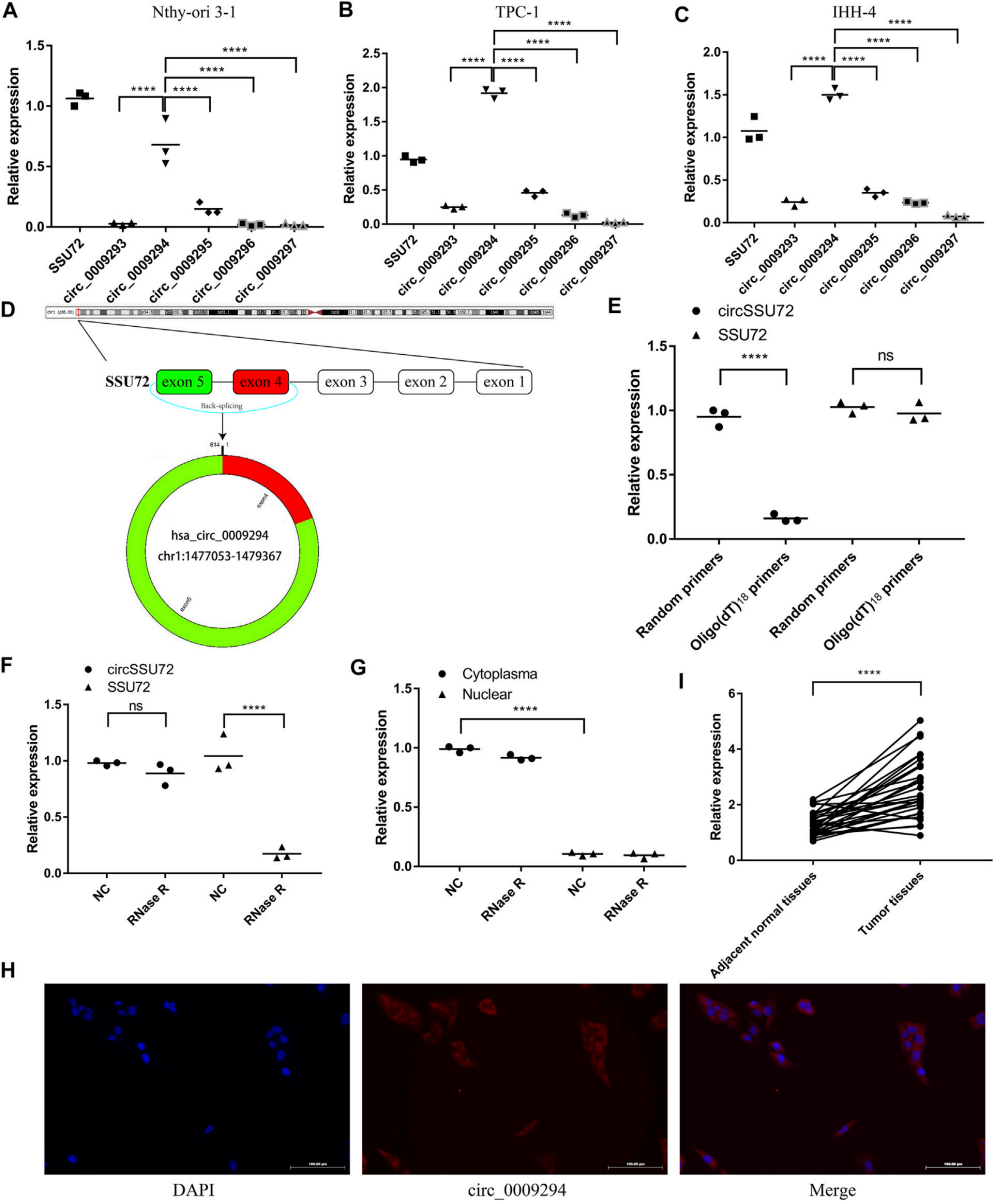
circSSU72 was upregulated in PTC **(A)** The expression of SSU72-related circRNAs in the Nthy-ori 3-1 cells (*n* = 3). **(B)** The expression of SSU72-related circRNAs in the TPC-1 cells (*n* = 3). **(C)** The expression of SSU72-related circRNAs in the IHH-4 cells (*n* = 3). **(D)** The diagram exhibiting the formation of circSSU72 (hsa_circ_0009294). **(E)** qRT-PCR detected the levels of circSSU72 and SSU72 mRNA after reverse transcribed with random primers and oligo (dT)_18_ primers (*n* = 3). **(F)** The relative expression of circSSU72 and SSU72 mRNA after treatment of RNase R (*n* = 3). **(G)** circSSU72 was separately detected in nuclear and cytoplasm (*n* = 3). **(H)** The expression level of circSSU72 (hsa_circ_0009294) was evaluated by FISH assay in Nthy-ori 3-1 cells. circSSU72 was stained red and nuclei were stained blue using 4ʹ,6-diamidino-2-phenylindole (DAPI). Scale bars = 100 µm. **(I)** The expression of circSSU72 was assessed by qRT-PCR assay in PTC tissues and adjacent normal tissues (*n* = 30). *p < 0.05; **p < 0.01; ***p < 0.001; ****p < 0.0001.

circRNAs derived from SSU72 were investigated in the CircBank Database, and 8 circRNAs were found. After excluding 3 circRNAs containing introns, 5 circRNAs derived from extrons were finally included in this study (hsa_circ_0009293, hsa_circ_0009294, hsa_circ_0009295, hsa_circ_0009296, hsa_circ_0009297). We subsequently confirmed the expression pattern of these SSU72-related circRNAs in Nthy-ori 3-1, TPC-1, IHH-4 cells by qRT-PCR assay with divergent primers (Figures 1A–C) Results showed that hsa_circ_0009294 dominated the SSU72-related circRNAs. Thus, we named hsa_circ_0009294 as circSSU72 and the following researches were focused on circSSU72 (Figure 1D).

As shown in Figure 1E, oligo (dT)_18_ primers were not able to achieve reverse transcription, indicating circSSU72 was a closed-loop structure. To confirm the cyclization and stability of circSSU72, an RNase R treatment was applied. The liner SSU72 mRNA decreased significantly, while the circSSU72 showed insensitivity to RNase R (Figure 1F). Meanwhile, nuclear and cytoplasmic RNA was extracted respectively, and qRT-PCR showed circSSU72 was mainly localized in the cytoplasm with insensitivity to RNase R (Figure 1G). The FISH assay also demonstrated that circSSU72 (hsa_circ_0009294) was expressed in the cytoplasm of Nthy-ori 3-1 cell (Figure 1H).

To determine the role of circSSU72 in PTC, the expression of circSSU72 was examined by qRT-PCR in 30 PTC patients. And the results showed circSSU72 was significantly upregulated in PTC tissues than adjacent normal tissues (Figure 1I). Patients were subsequently divided into the circSSU72-low group and the circSSU72-high group, and the patient and tumor characteristics were shown in Table 2. The higher level of circSSU72 was significantly associated with bigger lesions, capsule invasion, and lymph node metastasis.

**TABLE 2:** Patient and tumor characteristics.

Characteristics	circSSU72-low group (*n* = 15)	circSSU72-high group (*n* = 15)	*P*
Age (years)	41.13 ± 8.52	36.80 ± 8.21	0.167
Gender			1.000
Female	10 (66.7)	11 (73.3)	
Male	5 (33.3)	4 (26.7)	
Bilateral lesion			0.080
Yes	1 (6.7)	6 (40.0)	
No	14 (93.3)	9 (60.0)	
Largest tumor size (cm)	0.63 ± 0.81	1.26 ± 0.59	0.021
Number of lesion			0.064
Single	12 (80.0)	7 (46.7)	
Multiple	3 (20.0)	8 (53.3)	
Capsule invasion			0.042
Yes	0 (0.0)	5 (33.3)	
No	15 (100.0)	10 (66.7)	
Lymph node metastasis			0.008
Yes	2 (13.3)	10 (66.7)	
No	13 (86.7)	5 (33.3)	

Data are expressed as mean ± standard deviation or n (%).

### The Silence of circSSU72 Inhibited PTC Cell Proliferation, Migration, and Invasion

**FIGURE 2:**
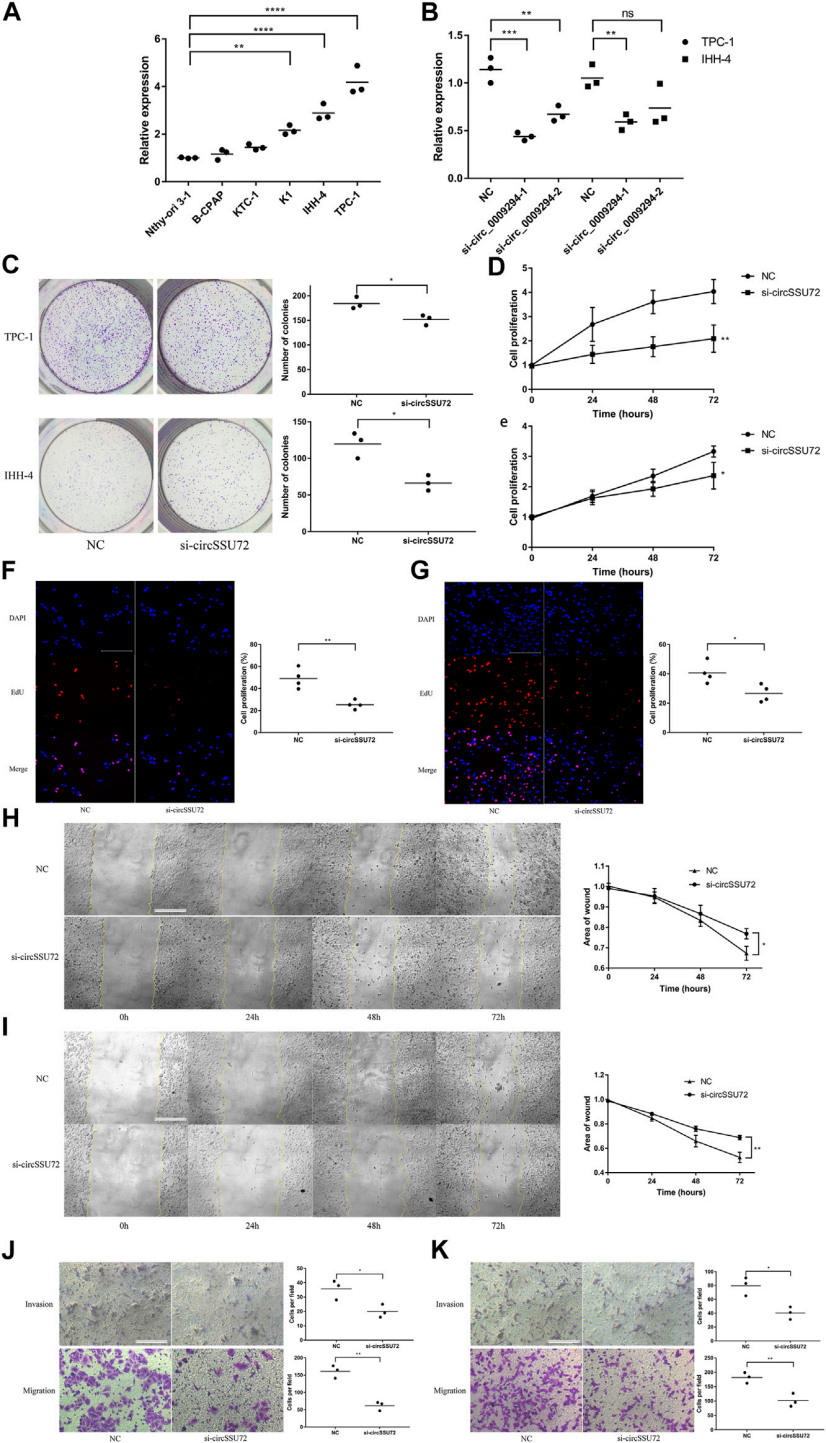
The silence of circSSU72 inhibited PTC cell proliferation, migration, and invasion. **(A)** The expression of circSSU72 was investigated in five PTC cell lines (*n* = 3). **(B)** TPC-1 and IHH-4 cells were transfected with siRNAs of circSSU72, and the expression level of circSSU72 was analyzed by qRT-PCR assay (*n* = 3). **(C)** Colony formation assay of the cell proliferation ability in TPC-1 and IHH-4 cells transfected with si-circSSU72 (*n* = 3). **(D)** CCK8 assay to assess the influence of circSSU72 on TPC-1 cell (*n* = 3). **(E)** CCK8 assay to assess the influence of circSSU72 on IHH-4 cell (*n* = 3). **(F)** EdU assay of the cell proliferation ability in TPC-1 cell transfected with si-circSSU72 (scale bar = 100 um, *n* = 4). **(G)** EdU assay of the cell proliferation ability in IHH-4 cell transfected with si-circSSU72 (scale bar = 100 um, *n* = 4). **(H)** Scratch wound healing assays in transfected TPC-1 cell (scale bar = 200 um, *n* = 3). **(I)** Scratch wound healing assays in transfected IHH-4 cell (scale bar = 200 um, *n* = 3). **(J)** Transwell invasion and migration assay in transfected TPC-1 cell (scale bar = 100 um, *n* = 3). **(K)** Transwell invasion and migration assay in transfected IHH-4 cell (scale bar = 100 um, *n* = 3). *p* < 0.05; **p < 0.01; ***p < 0.001; ****p < 0.0001.

The expression of circSSU72 was subsequently investigated in multiple cell lines. Among five PTC cell lines (B-CPAP, KTC-1, K1, IHH-4, TPC-1), TPC-1 and IHH-4 showed the highest level of circSSU72 expression (Figure 2A). To explore whether circSSU72 could affect the progression of PTC, TPC-1 and IHH-4 cells were transfected with circSSU72 siRNAs. As shown in Figure 2B, two siRNAs both decreased the expression of circSSU72 in TPC-1 cells, however there was no significant difference between the NC group and si-circ_0009294-2 in IHH-4 cells. Thus, si-circ_0009294-1 was chosen for further experiments. The clone formation (Figure 2C), CCK-8 assay (Figures 2D,E), and EdU assay (Figures 2F,G) revealed that the proliferation of PTC cells was inhibited in the si-circSSU72 group compared with the NC group. Meanwhile, wound healing assay (Figures 2H,I) and Transwell invasion and migration assay (Figures 2J,K) revealed the ability of invasion and migration of PTC cells was also inhibited by interfering circSSU72.

Meantime, circSSU72 overexpressing cell lines were also constructed by circSSU72 overexpression vectors using consistent cell lines (Supplementary Figure S1). CCK-8 assay, EdU assay, and Transwell invasion and migration assay confirmed that circSSU72 overexpressing promoted cell proliferation, invasion, migration in these two PTC cell lines (Supplementary Figure S1). Tumor growth was assayed *in vivo* to further investigate the roles of circSSU72. The volume and weight of tumors from circSSU72 overexpressing TPC-1 cells were significantly higher compared with tumors from NC TPC-1 cells (Supplementary Figure S1).

### circSSU72 Functioned as a Sponge for miR-451a

**FIGURE 3:**
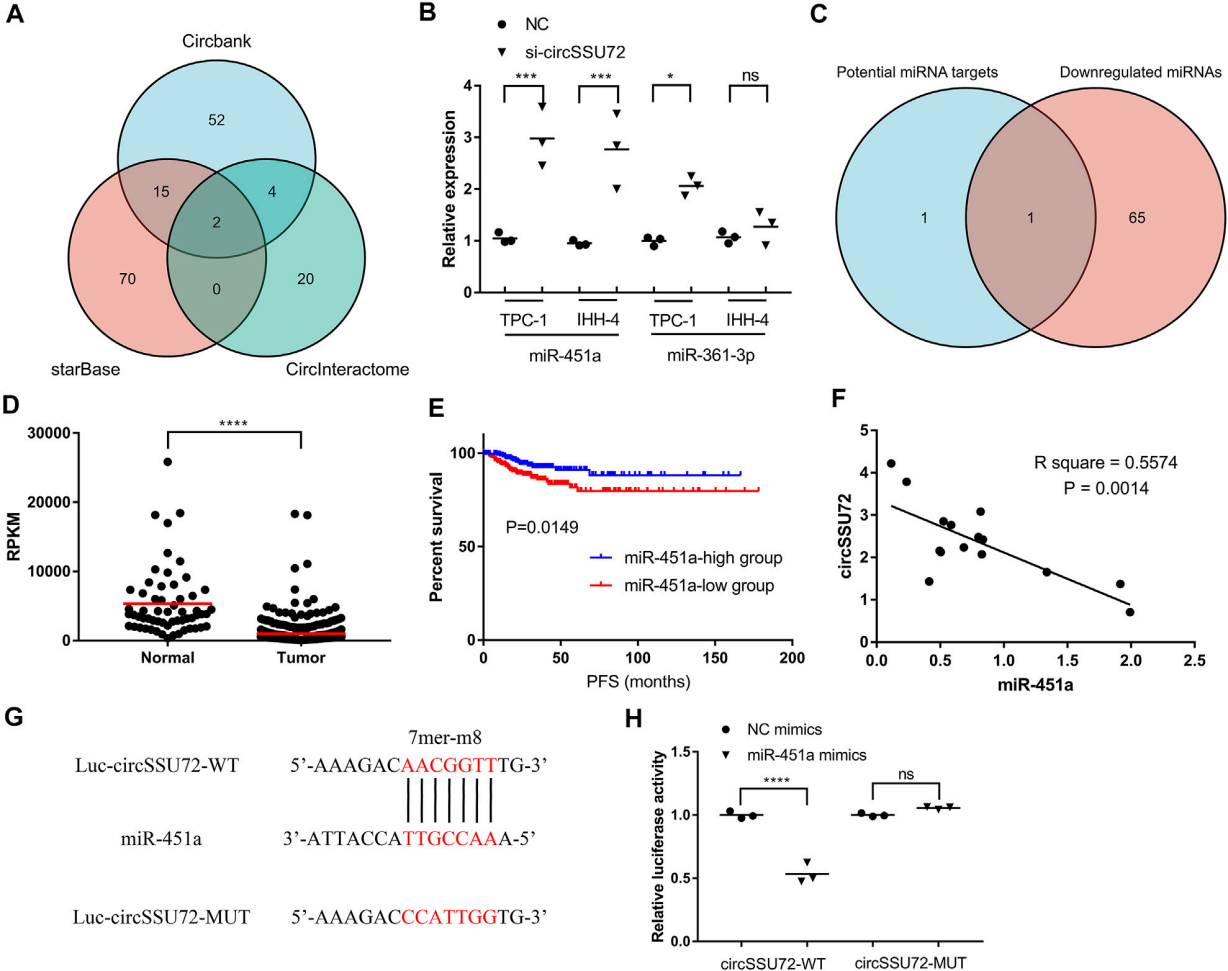
circSSU72 functioned as a sponge for miR-451a. **(A)** Venn plot of three databases, including Circbank, starBase, and CircInteractome, to predict the targets of circSSU72. **(B)** The expression of miR-361–3p and miR-451a in TPC-1 and IHH-4 cells with or without si-circSSU72 by qRT-PCR (*n* = 3). **(C)** Venn plot of potential target miRNAs and downregulated miRNAs in TCGA-THCA. **(D)** The expression of miR-451a in TCGA-THCA. **(E)** The Kaplan-Miere curve of miR-451a concerning the progression-free survival of TCGA-THCA. **(F)** Correlation between circSSU72 and miR-451a expression in 15 pairs PTC tissues (*n* = 15). **(G,H)** A putative interaction site with miR-451a in circSSU72 was predicted and verified by dual-luciferase reporter assay in TPC-1 cell (*n* = 3). *p < 0.05; **p < 0.01; ***p < 0.001; ****p < 0.0001.

Multiple studies have proven that circRNAs could function by sponging miRNAs. Therefore, we subsequently explored the potential miRNAs associated with circSSU72. Three databases, including Circbank, starBase, and CircInteractome, were used for the selection of circSSU72-associated miRNAs. The Venn plot showed two miRNAs (miR-361–3p, miR-451a) were potential targets of circSSU72 (Figure 3A). We further examined the expression of these two miRNAs with or without si-circSSU72 by qRT-PCR. miR-451a was significantly upregulated when inhibiting circSSU72 in both TPC-1 and IHH-4 cells, while the expression of miR-361–3p did not change significantly in IHH-4 cells (Figure 3B). Meanwhile, we also investigated miRSeq data of THCA patients in the TCGA database, and the intersection between potential miRNA targets and downregulated miRNAs with log_2_ (fold change) ≤-1 included only miR-451a (Figure 3C). The expression data of miR-451a in TCGA-THCA were shown in Figure 3D, and the Kaplan-Meier plot also showed that miR-451a was associated with better progression-free survival (Figure 3E), which was consistent with being a target of circSSU72. Furthermore, the circSSU72 expression was negatively associated with the miR-451a expression in PTC tissues (Figure 3F). Dual-luciferase reporters assay also validated the direct interaction between circSSU72 and miR-451a (Figures 3G,H).

### The Silence of circSSU72 Inhibited PTC Cell Proliferation, Migration, and Invasion by Targeting miR-451a

**FIGURE 4:**
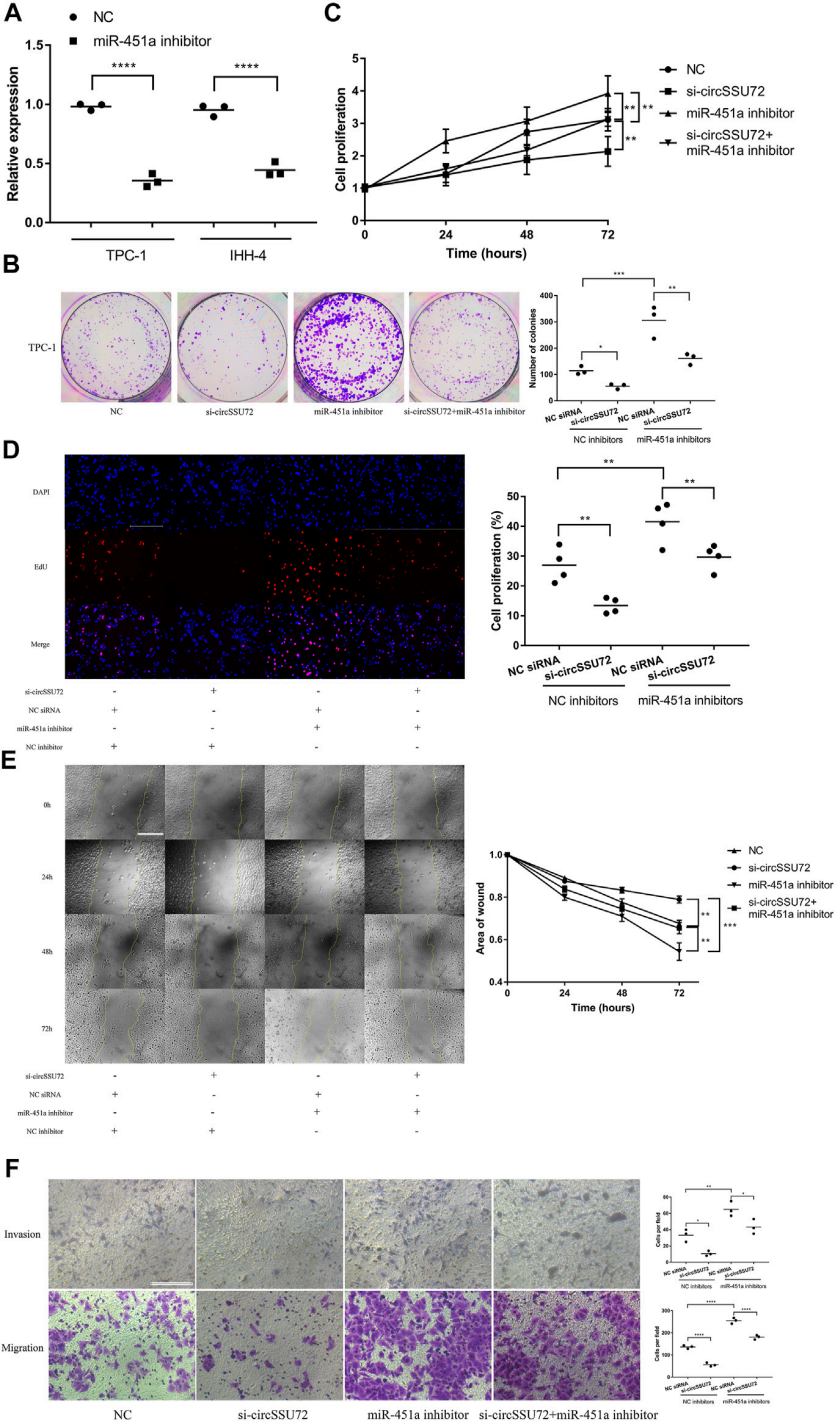
The silence of circSSU72 inhibited PTC cell proliferation, migration, and invasion by targeting miR-451a. **(A)** The expression of miR-451a in TPC-1 and IHH-4 cells with the transfection of miR-451a inhibitor (*n* = 3). **(B)** Colony formation assay of the cell proliferation ability in TPC-1 cell transfected with si-circSSU72 and miR-451a inhibitor (*n* = 3). **(C)** CCK8 assay to assess the influence of circSSU72 and miR-451a on TPC-1 cell (*n* = 3). **(D)** EdU assay of the cell proliferation ability in TPC-1 cell transfected with si-circSSU72 and miR-451a inhibitor (scale bar = 100 um, *n* = 4). **(E)** Scratch wound healing assays in transfected TPC-1 cell (scale bar = 200 um, *n* = 3). **(F)** Transwell invasion and migration assay in transfected TPC-1 cell (scale bar = 100 um, *n* = 3). *p < 0.05; **p < 0.01; ***p < 0.001; ****p < 0.0001.

miR-451a inhibitors were proven to be effective in both two PTC cells (Figure 4A), and further assays were performed to confirm the function of the circSSU72/miR-451a axis. The clone formation, CCK-8 assay, and EdU assay revealed that the proliferation of PTC cells was inhibited by si-circSSU72 and miR-451a inhibitor reversed the suppressive effects in TPC-1 (Figures 4B–D) and IHH-4 cells (Supplementary Figure S2). Meanwhile, wound healing assay and Transwell invasion and migration assay revealed the suppressive effects of si-circSSU72 on the ability of invasion and migration of TPC-1 (Figures 4E,F) and IHH-4 cells (Supplementary Figure S2) could also be reversed by miR-451a inhibiting. These results indicated that circSSU72 affected the proliferation, migration, and invasion of PTC cells by targeting miR-451a.

### miR-451a Targeted S1PR2 in PTC Cells

**FIGURE 5:**
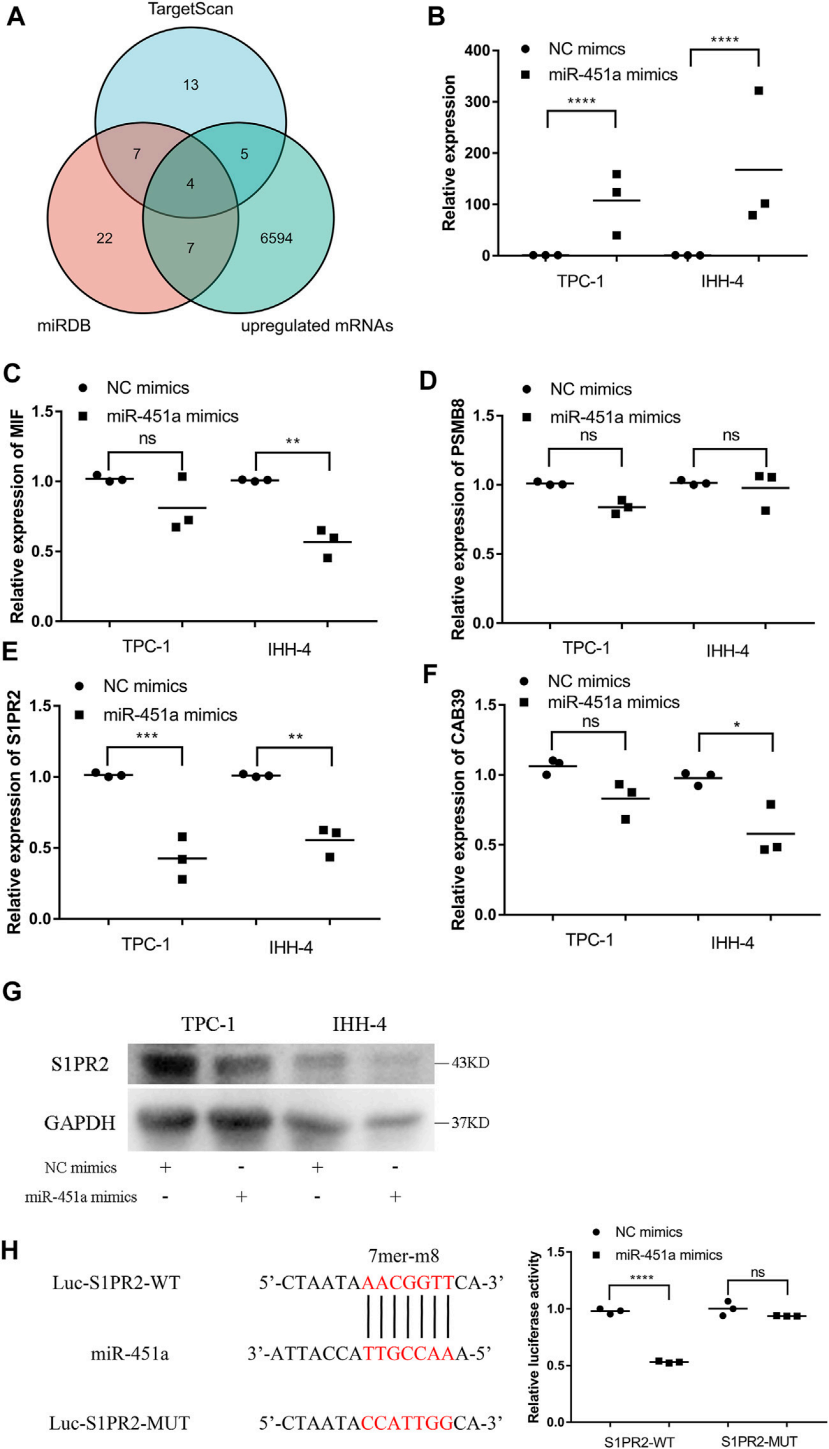
miR-451a targeted S1PR2 in PTC cells. **(A)** Venn plot of three databases, including TargetScan, miRDB, and upregulated mRNAs in TCGA-THCA. **(B)** The expression of miR-451a in TPC-1 and IHH-4 cells with the transfection of miR-451a mimics (*n* = 3). **(C)** The expression of MIF in TPC-1 and IHH-4 cells with or without miR-451a mimics by qRT-PCR (*n* = 3). **(D)** The expression of PSMB8 in TPC-1 and IHH-4 cells with or without miR-451a mimics by qRT-PCR (*n* = 3). **(E)** The expression of S1PR2 in TPC-1 and IHH-4 cells with or without miR-451a mimics by qRT-PCR (*n* = 3). **(F)** The expression of CAB39 in TPC-1 and IHH-4 cells with or without miR-451a mimics by qRT-PCR (*n* = 3). **(G)** The expression of S1PR2 protein in TPC-1 and IHH-4 cells with or without miR-451a mimics by western blot assay. **(H)** A putative interaction site with miR-451a in S1PR2 was predicted and verified by dual-luciferase reporter assay in TPC-1 cell (*n* = 3). *p < 0.05; **p < 0.01; ***p < 0.001; ****p < 0.0001.

To explore the potential target of miR-451a, TargetScan, miRDB, and transcriptome data from TCGA-THCA were used for predicting potential targets. As shown in Figure 5A 4 genes, including MIF (Macrophage Migration Inhibitory Factor), PSMB8 (Proteasome 20S Subunit Beta 8), S1PR2 (Sphingosine-1-Phosphate Receptor 2), and CAB39 (Calcium Binding Protein 39), were candidates for the target of miR-451a. miR-451a mimics were successfully transfected into PTC cells (Figure 5B), and qRT-PCR showed that only S1PR2 was significantly regulated after the transfection of miR-451a mimics in both TPC-1 and IHH-4 cells (Figures 5C–F). Subsequently, a western blot assay was performed to confirm the downregulation of S1PR2 by miR-451a mimics (Figure 5G). Dual-luciferase reporters assay also validated the direct interaction between miR-451a and S1PR2 (Figure 5H).

### miR-451a Inhibited the Proliferation, Migration, and Invasion of PTC Cells by Targeting S1PR2

**FIGURE 6:**
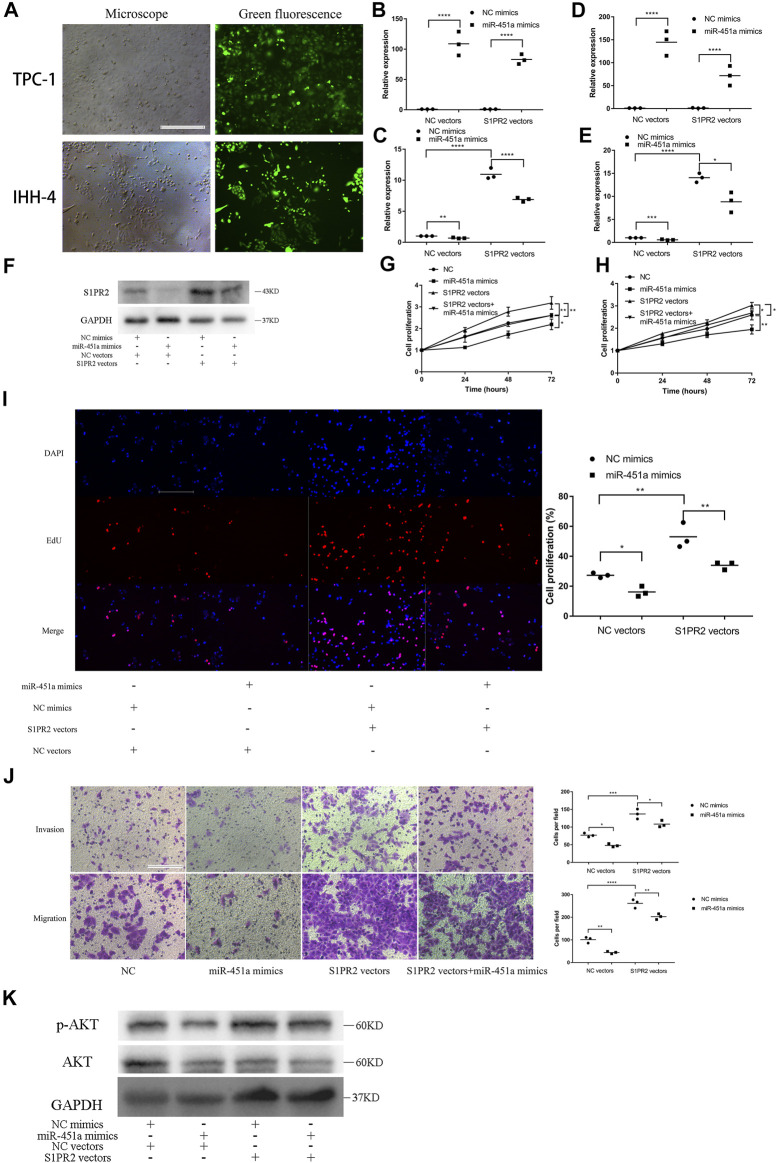
miR-451a inhibited the proliferation, migration, and invasion of PTC cells by targeting S1PR2. **(A)** Green fluorescent showing the successful transfection of S1PR2 overexpression vectors in TPC-1 and IHH-4 cells (scale bar = 200 um). **(B)** The efficacy of miR-451a mimics was reconfirmed in TPC-1 cell with the transfection of S1PR2 vectors and NC vectors (*n* = 3). **(C)** The expression of S1PR2 in TPC-1 cell with the transfection of S1PR2 vectors miR-451a mimics (*n* = 3). **(D)** The efficacy of miR-451a mimics was reconfirmed in IHH-4 cell with the transfection of S1PR2 vectors and NC vectors (*n* = 3). **(E)** The expression of S1PR2 in IHH-4 cell with the transfection of S1PR2 vectors miR-451a mimics (*n* = 3). **(F)** The expression of S1PR2 protein in transfected TPC-1 cell by western blot assay. **(G)** CCK8 assay to assess the influence of miR-451a and S1PR2 on TPC-1 cell (*n* = 3). **(H)** CCK8 assay to assess the influence of miR-451a and S1PR2 on IHH-4 cell (*n* = 3). **(I)** EdU assay of the cell proliferation ability in TPC-1 cell transfected with miR-451a mimics and S1PR2 vectors (scale bar = 100 um, *n* = 3). **(J)** Transwell invasion and migration assay in transfected TPC-1 cell (scale bar = 100 um, *n* = 3). **(K)** The expression of AKT, p-AKT proteins in transfected TPC-1 cell by western blot assay.*p < 0.05; **p < 0.01; ***p < 0.001; ****p < 0.0001.

TPC-1 and IHH-4 cells were transfected with S1PR2 overexpression vectors or NC vectors. Green fluorescence showed that the transfections were successful (Figure 6A). The efficacy of miR-451a mimics and the expression of S1PR2 were also validated in TPC-1 (Figures 6B,C) and IHH-4 cells (Figures 6D,E). Subsequently, western blot assay was performed to confirm the overexpression of S1PR2 and the downregulation of S1PR2 by miR-451a mimics in TPC-1 cells (Figure 6F). CCK-8, EdU, Transwell invasion and migration assay indicated that S1PR2 reversed the inhibition effects of miR-451a on cell proliferation, migration, and invasion in TPC-1 (Figures 6G,I,J) and IHH-4 (Figure 6H; Supplementary Figure S3) cells, indicating that the effects of miR-451a on thyroid cancer cell proliferation, migration, and invasion depended on S1PR2 suppression.

To explore the potential pathway that circSSU72/miR451a/S1PR2 axis is involved in, the phosphorylation level of AKT was further investigated by western blot assay. And the results showed that the AKT pathway was activated by circSSU72/miR451a/S1PR2 axis (Figure 6K).

## Discussion

In this study, we comprehensively investigated the role of circRNAs derived from SSU72 in PTC, and further explored the mechanisms of circSSU72. We firstly found that circSSU72 (hsa_circ_0009294)/miR-451a/S1PR2 axis could regulate cell proliferation, migration, and invasion of PTC cells by the AKT pathway, which could serve as a novel therapeutic target in PTC.

The majority of circRNAs derive from the exons of protein-coding genes through back-splicing, while some circRNAs contain introns. (Starke et al., 2015) When spliceosomes or canonical pre-mRNA processing events are dysregulated, circRNAs may be the preferred output gene type. (Liang et al., 2017) Many studies have indicated that circRNAs are involved in the initiation and progression of multiple systematic diseases, cancers, etc., including thyroid cancer. (Xia et al., 2021) Since 2018, many circRNAs were found to be associated with the proliferation, migration, and invasion of thyroid cancer cells. (Chen et al., 2018; Cai et al., 2019) Recently, circTP53 was found to promote thyroid cancer cell proliferation by targeting miR-1233–3p/MDM2 axis. (Ma et al., 2021) Meanwhile, circRNA_102002 was found to facilitate metastasis of papillary thyroid cancer through regulating the miR-488–3p/HAS2 axis. To our knowledge, this was the first study reporting the role of circSSU72 (hsa_circ_0009294) in the development of thyroid cancer.

miRNAs are well-studied rich ncRNAs without coding protein. miR-451a was found to be a tumor suppressor in lung cancer, (Shen et al., 2018) colorectal cancer, (Xu et al., 2019) and prostate cancer. (Liu et al., 2020) In thyroid cancer, it has also been reported that miR-451a inhibited cell proliferation, migration, and invasion. Fan et al. reported that miR-451a could inhibit proliferation, epithelial-mesenchymal transition, and induce apoptosis in PTC cells. (Fan and Zhao, 2019) Moreover, Wang et al. also reported that miR-451a restrained the growth and metastatic phenotypes of PTC cells through targeting ZEB1. (Wang et al., 2020) In this study, we confirmed the tumor-suppressive role of miR-451a in PTC cell proliferation, migration, and invasion. Moreover, we also found that miR-451 might function through regulating S1PR2.

S1PRs are G protein-coupled receptors, which regulate various functions, including cell survival and growth, migration, and cytoskeleton organization. (Aarthi et al., 2011) S1PR2 is located on the plasma membrane and in the cytoplasm of mammalian cells, and couples to members of the G_i_, G_q_, and G_12/13_ families. (Yu, 2021) Furthermore, S1PR2 was reported to be associated with various cancers. Yin et al. reported that S1PR2 was involved in the growth of hepatocellular carcinoma cells, (Yin et al., 2018) while Pang et al. demonstrated that the knockdown of S1PR2 might contribute to the initial extramedullary translocation by promoting myeloma cell migration and invasion through NF-κB pathway activation. (Pang et al., 2020) More recently, S1PR2 was reported to contribute to the growth of hepatocellular carcinoma through the AKT pathway. (Yin et al., 2018) In the present study, we firstly found that over-expressing S1PR2 might promote the abilities of proliferation, migration, and invasion of PTC cells.

Different behaviors were shown in the mechanism mediated by miR-451a between IHH-4 cells and TPC-1 cells. Although the miR-451a/S1PR2 axis was universal in these PTC cell lines, the BRAF gene mutation may cause the different behaviors since the IHH-4 involves BRAF gene mutation while the TPC-1 do not. Future studies should focus on the associations between the mechanisms mediated by miR-451a and BRAF gene mutation.

This was a pre-clinical study with certainly some limitations. A large cohort of thyroid cancer patients is needed to validate the expression pattern of circSSU72. Meanwhile, the relationships between the circSSU72 and its parental mRNA SSU72 should be comprehensively investigated in future studies. Lastly, *in vivo* studies will be performed in the near future to prompt the translation from experimental discoveries to clinical practices of circSSU72-related therapies.

## Conclusion

The expression of circSSU72 (hsa_circ_0009294) increases in PTC. The inhibition of circSSU72 is shown to suppress cell proliferation, migration, and invasion of PTC cells by regulating the miR-451a/S1PR2 axis. The circSSU72/miR-451a/S1PR2 axis may regulate cell proliferation, migration, and invasion of PTC.

## Data Availability

The original contributions presented in the study are included in the article/Supplementary Material, further inquiries can be directed to the corresponding author.

## Supplementary Material

Supplementary Figure S1 The overexpression of circSSU72 promoted PTC cell proliferation, migration, and invasion. (a) TPC-1 and IHH-4 cells were transfected with circSSU72 overexpressing vectors or NC vectors, and the expression level of circSSU72 was analyzed by qRT-PCR assay (n=3). (b) CCK8 assay to assess the influence of circSSU72 on TPC-1 cell (n=3). (c) CCK8 assay to assess the influence of circSSU72 on IHH-4 cell (n=3). (d) EdU assay of the cell proliferation ability in TPC-1 cell transfected with oe-circSSU72 (n=3). (e) EdU assay of the cell proliferation ability in IHH-4 cell transfected with oe-circSSU72 (n=3). (f) Transwell invasion and migration assay in transfected TPC-1 cell (n=3). (g) Transwell invasion and migration assay in transfected IHH-4 cell (n=3). (h) Tumors after 4 weeks post-injection. (i) Tumor volume. (j) Tumor weight.*P<0.05; **P<0.01;***P<0.001;****P<0.0001.

Supplementary Figure S2 The silence of circSSU72 inhibited PTC cell proliferation, migration, and invasion by targeting miR-451a. (a) Colony formation assay of the cell proliferation ability in IHH-4 cell transfected with si-circSSU72 and miR-451a inhibitor (n=3). (b) CCK8 assay to assess the influence of circSSU72 and miR-451a on IHH-4 cell (n=3). (c) EdU assay of the cell proliferation ability in IHH-4 cell transfected with si-circSSU72 and miR-451a inhibitor (scale bar=100um, n=4). (d) Scratch wound healing assays in transfected IHH-4 cell (scale bar=200um, n=3). (e) Transwell invasion and migration assay in transfected IHH-4 cell (scale bar=100um, n=3).*P<0.05; **P<0.01;***P<0.001;****P<0.0001.

Supplementary Figure S3 miR-451a inhibited the proliferation, migration, and invasion of PTC cells by targeting S1PR2. (a) EdU assay of the cell proliferation ability in IHH-4 cell transfected with miR-451a mimics and S1PR2 vectors (scale bar=100um, n=3). (b) Transwell invasion and migration assay in transfected IHH-4 cell (scale bar=100um, n=3).*P<0.05; **P<0.01;***P<0.001;****P<0.0001.

## Abbreviations

ANOVA, One-way analysis of variance; circRNAs, circular RNAs; ceRNAs, competitive endogenous RNAs; CCK8, cell counting kit-8; DMEM, Dulbecco’s modifed Eagle’s medium; EdU, 5-Ethynyl-20-deoxyuridine; FBS, fetal bovine serum; miRNAs, micro RNAs; PTC, papillary thyroid carcinoma; TC, thyroid cancer.

## References

[B1] Aarthi J. J. Darendeliler M. A. Pushparaj P. N. (2011). Dissecting the Role of the S1P/S1PR axis in Health and Disease. J. Dent. Res. 90 (7), 841–854. 10.1177/0022034510389178 21248363

[B2] Agarwal V. Bell G. W. Nam J. W. Bartel D. P. (2015). Predicting Effective microRNA Target Sites in Mammalian mRNAs. Elife 4, e05005. 10.7554/eLife.05005 PMC4532895 26267216

[B3] Cai X. Zhao Z. Dong J. Lv Q. Yun B. Liu J. (2019). Circular RNA circBACH2 Plays a Role in Papillary Thyroid Carcinoma by Sponging miR-139-5p and Regulating LMO4 Expression. Cell Death Dis. 10 (3), 184. 10.1038/s41419-019-1439-y 30796202 PMC6385235

[B4] Chen F. Feng Z. Zhu J. Liu P. Yang C. Huang R. (2018). Emerging Roles of circRNA_NEK6 Targeting miR-370-3p in the Proliferation and Invasion of Thyroid Cancer via Wnt Signaling Pathway. Cancer Biol. Ther. 19 (12), 1139–1152. 10.1080/15384047.2018.1480888 30207869 PMC6301817

[B5] Chen Y. Wang X. (2020). miRDB: an Online Database for Prediction of Functional microRNA Targets. Nucleic Acids Res. 48 (D1), D127–D131. 10.1093/nar/gkz757 31504780 PMC6943051

[B6] DeGroot L. J. Kaplan E. L. McCormick M. Straus F. H. (1990). Natural History, Treatment, and Course of Papillary Thyroid Carcinoma*. J. Clin. Endocrinol. Metab. 71 (2), 414–424. 10.1210/jcem-71-2-414 2380337

[B7] Dudekula D. B. Panda A. C. Grammatikakis I. De S. Abdelmohsen K. Gorospe M. (2016). CircInteractome: A Web Tool for Exploring Circular RNAs and Their Interacting Proteins and microRNAs. RNA Biol. 13 (1), 34–42. 10.1080/15476286.2015.1128065 26669964 PMC4829301

[B8] Fagerberg L. Hallström B. M. Oksvold P. Kampf C. Djureinovic D. Odeberg J. (2014). Analysis of the Human Tissue-specific Expression by Genome-wide Integration of Transcriptomics and Antibody-Based Proteomics. Mol. Cell Proteomics 13 (2), 397–406. 10.1074/mcp.m113.035600 24309898 PMC3916642

[B9] Fan X. Zhao Y. (2019). miR‐451a Inhibits Cancer Growth, Epithelial‐mesenchymal Transition and Induces Apoptosis in Papillary Thyroid Cancer by Targeting PSMB8. J. Cel. Mol. Med. 23 (12), 8067–8075. 10.1111/jcmm.14673 PMC6850967 31559672

[B10] Hwang S. Kim M. Lee C. (2021). Ssu72 Dual-specific Protein Phosphatase: From Gene to Diseases. Int. J. Mol. Sci. 22 (7), 3791. 10.3390/ijms22073791 33917542 PMC8038829

[B11] Kim J. Gosnell J. E. Roman S. A. (2020). Geographic Influences in the Global Rise of Thyroid Cancer. Nat. Rev. Endocrinol. 16 (1), 17–29. 10.1038/s41574-019-0263-x 31616074

[B12] Laha D. Nilubol N. Boufraqech M. (2020). New Therapies for Advanced Thyroid Cancer. Front. Endocrinol. 11, 82. 10.3389/fendo.2020.00082 PMC7257776 32528402

[B13] Li J. H. Liu S. Zhou H. Qu L. H. Yang J. H. (2014). starBase v2.0: Decoding miRNA-ceRNA, miRNA-ncRNA and Protein-RNA Interaction Networks from Large-Scale CLIP-Seq Data. Nucleic Acids Res. 42 (Database issue), D92–D97. 10.1093/nar/gkt1248 24297251 PMC3964941

[B14] Liang D. Tatomer D. C. Luo Z. Wu H. Yang L. Chen L. L. (2017). The Output of Protein-Coding Genes Shifts to Circular RNAs when the Pre-mRNA Processing Machinery Is Limiting. Mol. Cel. 68 (5), 940–e3. 10.1016/j.molcel.2017.10.034 PMC5728686 29174924

[B15] Liu M. Wang Q. Shen J. Yang B. B. Ding X. (2019). Circbank: a Comprehensive Database for circRNA with Standard Nomenclature. RNA Biol. 16 (7), 899–905. 10.1080/15476286.2019.1600395 31023147 PMC6546381

[B16] Liu Y. Yang H. Z. Jiang Y. J. Xu L. Q. (2020). miR‐451a Is Downregulated and Targets PSMB8 in Prostate Cancer. Kaohsiung J. Med. Sci. 36 (7), 494–500. 10.1002/kjm2.12196 32128987 PMC11896265

[B17] Ma W. Zhao P. Zang L. Zhang K. Liao H. Hu Z. (2021). CircTP53 Promotes the Proliferation of Thyroid Cancer via Targeting miR-1233-3p/MDM2 axis. J. Endocrinol. Invest. 44 (2), 353–362. 10.1007/s40618-020-01317-2 32500444

[B18] Pang M. Li C. Zheng D. Wang Y. Wang J. Zhang W. (2020). S1PR2 Knockdown Promotes Migration and Invasion in Multiple Myeloma Cells via NF-Κb Activation. Cmar Vol. 12, 7857–7865. 10.2147/cmar.s237330 PMC7457837 32922084

[B19] Rodríguez-Torres A. M. Lamas-Maceiras M. García-Díaz R. Freire-Picos M. A. (2013). Structurally Conserved and Functionally Divergent Yeast Ssu72 Phosphatases. Febs Lett. 587 (16), 2617–2622. 10.1016/j.febslet.2013.06.044 23831060

[B20] Sebastian S. O. Gonzalez J. M. Paricio P. P. Perez J. S. Flores D. P. Madrona A. P. (2000). Papillary Thyroid Carcinoma: Prognostic index for Survival Including the Histological Variety. Arch. Surg. 135 (3), 272–277. 10.1001/archsurg.135.3.272 10722027

[B21] Shen Y. Y. Cui J. Y. Yuan J. Wang X. (2018). MiR-451a Suppressed Cell Migration and Invasion in Non-small Cell Lung Cancer through Targeting ATF2. Eur. Rev. Med. Pharmacol. Sci. 22 (17), 5554–5561. 10.26355/eurrev_201809_15818 30229828

[B22] Starke S. Jost I. Rossbach O. Schneider T. Schreiner S. Hung L.-H. (2015). Exon Circularization Requires Canonical Splice Signals. Cel. Rep. 10 (1), 103–111. 10.1016/j.celrep.2014.12.002 25543144

[B23] Wang Q. Shang J. Zhang Y. Zhou Y. Tang L. (2020). MiR-451a Restrains the Growth and Metastatic Phenotypes of Papillary Thyroid Carcinoma Cells via Inhibiting ZEB1. Biomed. Pharmacother. 127, 109901. 10.1016/j.biopha.2020.109901 32335297

[B24] Xia F. Chen Y. Jiang B. Bai N. Li X. (2020). Hsa_circ_0011385 Accelerates the Progression of Thyroid Cancer by Targeting miR-361-3p. Cancer Cel. Int. 20, 49. 10.1186/s12935-020-1120-7 PMC7017482 32082079

[B25] Xia F. Zhang Z. Li X. (2021). Emerging Roles of Circular RNAs in Thyroid Cancer. Front. Cel. Dev. Biol. 9, 636838. 10.3389/fcell.2021.636838 PMC8107370 33981702

[B26] Xu K. Han B. Bai Y. Ma X.-Y. Ji Z.-N. Xiong Y. (2019). MiR-451a Suppressing BAP31 Can Inhibit Proliferation and Increase Apoptosis through Inducing ER Stress in Colorectal Cancer. Cel. Death Dis. 10 (3), 152. 10.1038/s41419-019-1403-x PMC6377610 30770794

[B27] Yin Y. Xu M. Gao J. Li M. (2018). Alkaline Ceramidase 3 Promotes Growth of Hepatocellular Carcinoma Cells via Regulating S1P/S1PR2/PI3K/AKT Signaling. Pathol. - Res. Pract. 214 (9), 1381–1387. 10.1016/j.prp.2018.07.029 30097213

[B28] Yu H. (2021). Targeting S1PRs as a Therapeutic Strategy for Inflammatory Bone Loss Diseases-Beyond Regulating S1P Signaling. Ijms 22 (9), 4411. 10.3390/ijms22094411 33922596 PMC8122917

