# Supplementary figures and images for: circSSU72 Promotes Cell Proliferation, Migration and Invasion of Papillary Thyroid Carcinoma Cells by Targeting miR-451a/S1PR2 Axis
Source: Front Cell Dev Biol. 2022 Mar 14;10:817028. doi: 10.3389/fcell.2022.817028 (PMC8967131; doi:10.3389/fcell.2022.817028)

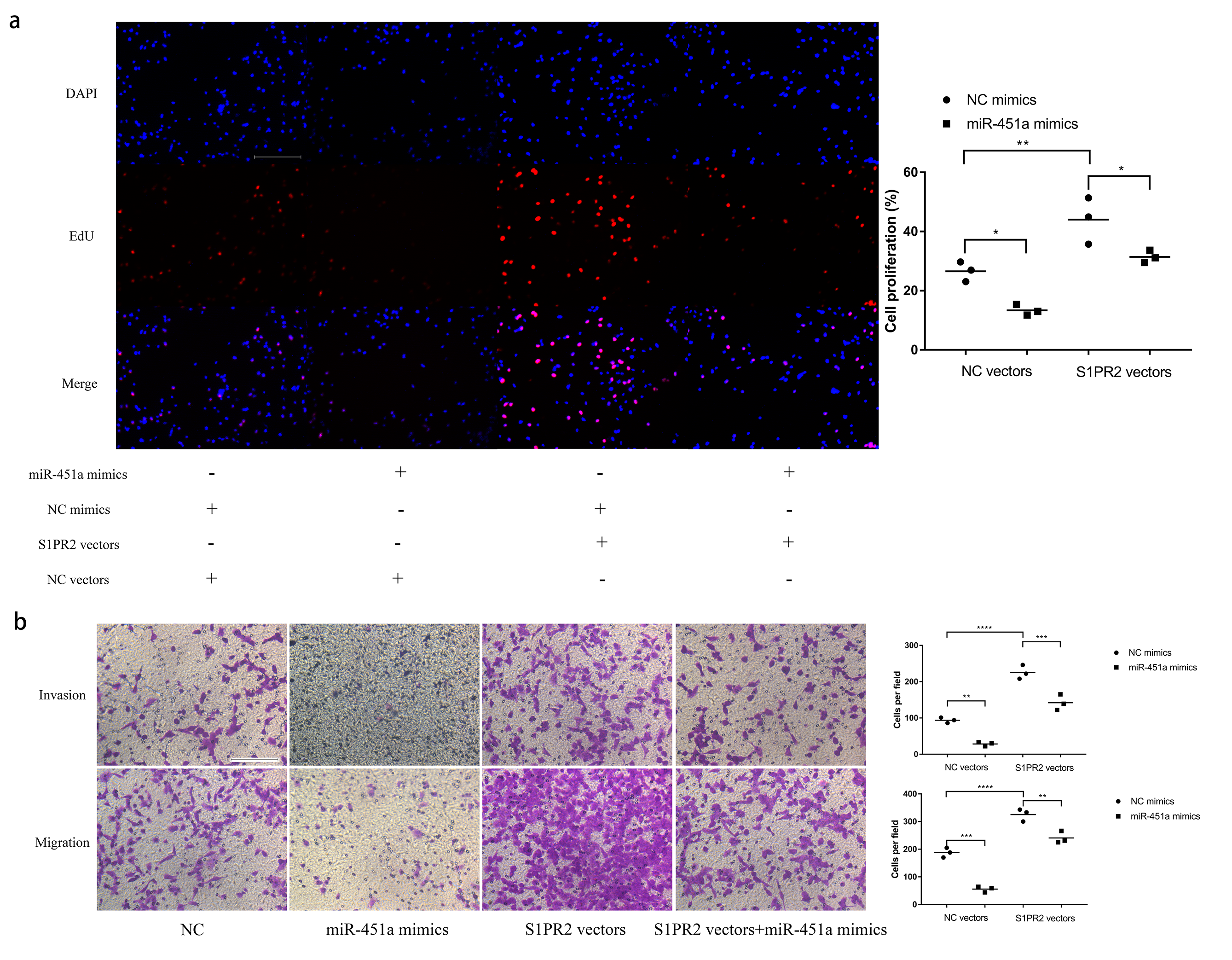

Supplement: Supplementary file 1 [file Image3.TIF]

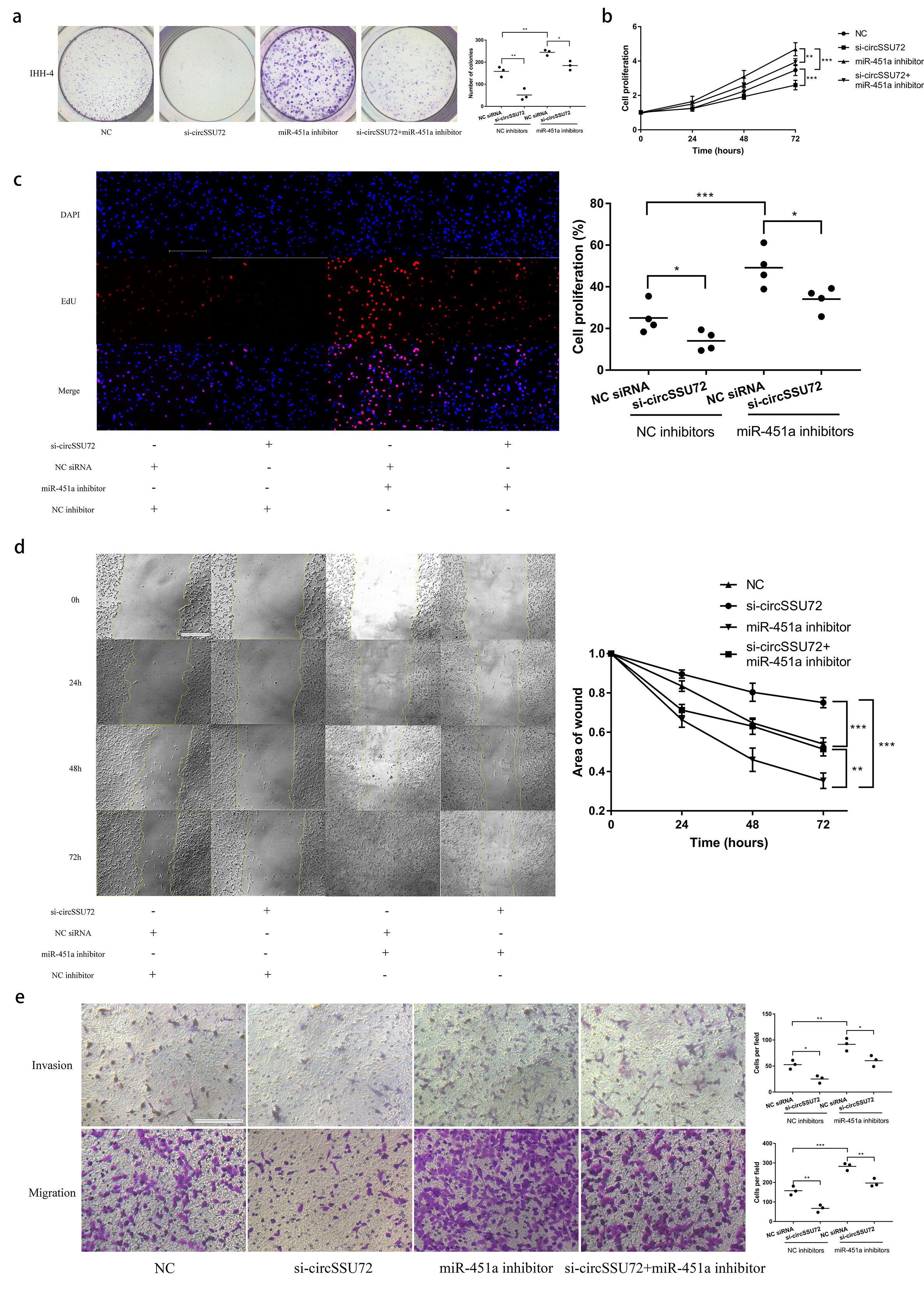

Supplement: Supplementary file 2 [file Image2.TIF]

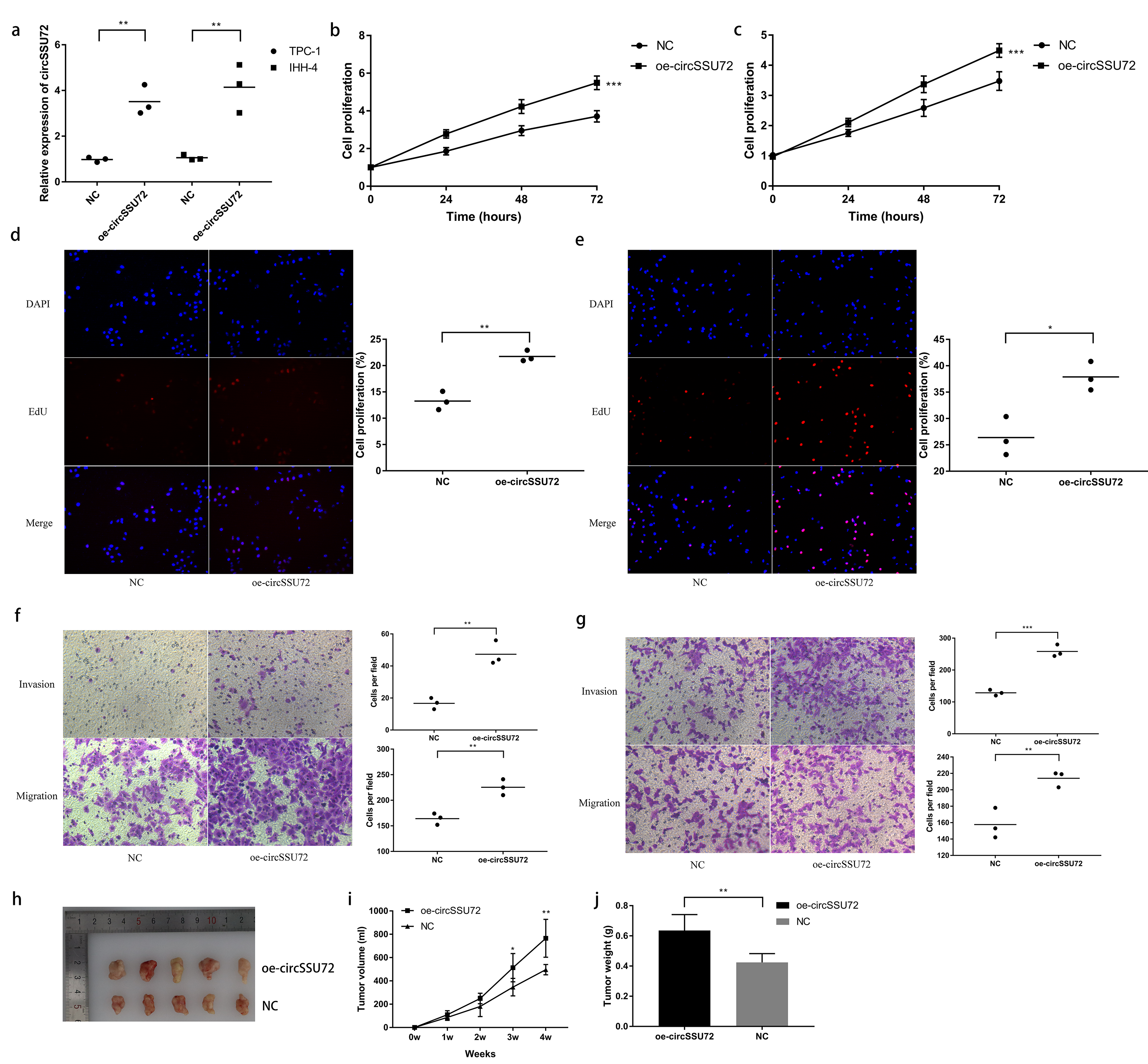

Supplement: Supplementary file 3 [file Image1.TIF]
